# Infiltrated bunch of solitons in Bi-doped frequency-shifted feedback fibre laser operated at 1450 nm

**DOI:** 10.1038/srep44194

**Published:** 2017-03-10

**Authors:** Joona Rissanen, Dmitry A. Korobko, Igor O. Zolotovsky, Mikhail Melkumov, Vladimir F. Khopin, Regina Gumenyuk

**Affiliations:** 1 Optoelectronics Research Centre, Tampere University of Technology, 3 Korkeakoulunkatu, 33720 Tampere, Finland; 2 Ulyanovsk State University, 42 Leo Tolstoy street, 432017, Ulyanovsk, Russia; 3 Fiber Optics Research Center, Russian Academy of Sciences, 38 Vavilov Street, 119333 Moscow, Russia; 4 Institute of Chemistry of High-Purity Substances, Russian Academy of Sciences, 49 Tropinin Street, 603600 Nizhny Novgorod, Russia

## Abstract

Mode-locked fibre laser as a dissipative system is characterized by rich forms of soliton interaction, which take place via internal energy exchange through noisy background in the presence of dispersion and nonlinearity. The result of soliton interaction was either stationary-localized or chaotically-oscillated soliton complexes, which have been shown before as stand-alone in the cavity. Here we report on a new form of solitons complex observed in Bi-doped mode-locked fibre laser operated at 1450 nm. The solitons are arranged in two different group types contemporizing in the cavity: one pulse group propagates as bound solitons with fixed phase relation and interpulse position eventuated in 30 dB spectrum modulation depth; while the other pulses form a bunch with continuously and chaotically moving solitons. The article describes both experimental and theoretical considerations of this effect.

Soliton fibre lasers display numerous intriguing multi-pulse dynamics, such as bound solitons or soliton molecules^1^,^2^,^3^,^4^,^5^,^6^, soliton bunching^7^,^8^,^9^, and soliton rain^10^,^11^,^12^, which are commonly perceived as distinct types of laser operation. Multi-pulsing in general arises from soliton energy quantization and is characteristic to all fibre lasers with net anomalous cavity dispersion; nevertheless, the exact nature of soliton dynamics can vary greatly between different laser configurations. In ordinary or harmonic multi-pulse mode-locking, the solitons feel no significant attractive force towards each other and thus the pulses and the energy are relatively evenly distributed over the entire cavity. However, if such an attractive force exists, solitons can be confined to a narrow time window of up to several nanoseconds in duration as multi-soliton complexes (bound solitons, soliton bunches or soliton rain). A tightly bound soliton state with a short, stable interpulse distance and a fixed phase difference is formed of two or more solitons by direct soliton-soliton interaction via overlapping pulse profiles. In contrast, a soliton bunch, which is a collection of constantly moving solitons with random separations and relative phases, can be the result of a weak, phase-insensitive, attractive force created by a saturable absorber with slow recovery time^7^,^8^. Soliton rain, on the other hand, is a more complex phenomenon which involves the constant generation of individual solitons from an unstable continuous-wave (CW) background and the drifting of the generated solitons towards a soliton cluster that also exists in the cavity.

These different aspects of multi-soliton dynamics have been intensively studied using mostly erbium-doped active fibre but with a variety of different mode-locking mechanisms. After the theoretical prediction of bound solitons by Malomed in 1991^13^, they were first experimentally observed in ring-cavity fibre lasers mode-locked with nonlinear polarization rotation^14^,^15^. Important later demonstrations of bound solitons include a figure-of-eight fibre laser mode-locked with a nonlinear optical loop mirror^16^ and in a ring laser with a carbon nanotube saturable absorber^1^. Soliton bunches in turn have been observed using semiconductor saturable absorber mirror^7^ and graphene^17^ absorbers, which had bi-exponential recovery mechanisms in contrast to fast nonlinearity-based mode-locking techniques. Soliton rain was first demonstrated in a ring cavity with nonlinear polarization rotation mode-locking by Chouli S. *et al*.^10^ and more recently using a nonlinear optical loop mirror^18^.

In terms of potential applications, multi-pulse dynamics in anomalous dispersion fibre cavities has mostly been the subject of purely academic interest. However, as recently demonstrated, short bursts of low-energy pulses do have advantages over single high-energy pulses in ablation-cooled material removal of biological tissues^19^. In particular, harmful nonlinear effects are reduced because the peak power is lower for pulse bursts. Bunched soliton fibre lasers naturally produce short bursts of pulses that can be amplified in a separate amplifier; therefore, it is conceivable that they could find practical uses in materials processing in the future. In bound soliton fibre lasers, the number of pulses in the burst is usually significantly lower than in bunched soliton lasers and, consequently, bound soliton lasers are less likely to find any high-power applications. However, the deep spectral modulation that arises from the phase-locking of bound solitons could make such lasers useful in wavelength division multiplexing. The further research on soliton complex formation allows to expect that the area of applications of multi-pulse soliton lasers can be greatly expanded, especially into metrology and sensing.

In recent years, the studies have been made on the pulse interaction and bound state formation in so-called hybrid mode-locked lasers, in which active and passive mode-locking elements are utilized simultaneously^20^,^21^,^22^. These studies indicate that the parameters of the generated soliton groups can be controlled within certain limits by introducing active elements, such as modulators and filters, in the cavity. In the current paper, we continue this research and report on the multi-pulse dynamics of a Bi-doped soliton fibre laser mode-locked with frequency-shifted feedback (FSF) technique^23^ at 1450 nm. The most important feature of the laser is that in addition to pure bound solitons and soliton bunches that are known to the literature it can also generate a combination of the two: an assembly where some of the solitons have fixed, constant separations and others are moving randomly.

## Results

### Experimental setup

**Figure 1:**
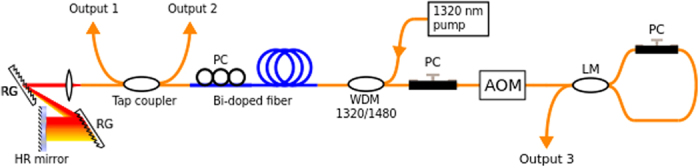
Bi-doped FSF fibre laser setup. AOM – acousto-optic modulator, WDM – wavelength division multiplexer, PC – polarization controller, LM – loop mirror, RG – reflection grating, HR mirror – high reflective mirror.

The fibre laser had the linear cavity configuration shown in Fig. 1. The total single-pass cavity length was 11.3 m of fibre and 0.2 m of free space with corresponding fundamental repetition rate of 9 MHz. The Bi-doped active fibre had a length of 4.0 m and was the same that was used in earlier research^24^; the rest of the fibre (7.3 m) was standard Corning SMF-28 single-mode telecom fibre. The laser was pumped through a wavelength division multiplexer using a semiconductor disk laser at 1320 nm with a maximum pump power of 1.6 W.

To mode-lock the laser with frequency shifted feedback technique, we used a fibre-coupled acousto-optic frequency shifter (MT80-IIR30-Fio-SMF by AA Optoelectronic) with +80 MHz frequency upshift, 2.2 dB insertion loss and ~0.4 dB polarization-dependent loss. Consequently, the frequency shift totalled 160 MHz in one round trip. The frequency shifter contained a highly birefringent 15 mm long TeO_2_ crystal with refractive indexes of 2.184 (fast axis) and 2.324 (slow axis) at 1450 nm. Because of this polarization-dependence, we used three polarization controllers in positions indicated by the figure. The frequency shifter guaranteed self-starting mode-locking, but the threshold pump power depended heavily on the state of the polarization controllers and varied roughly from 0.6 W to 1.5 W. Once mode-locking had started the pump power could be decreased to as low as 0.4 W without transition to CW operation. All measurements were taken via output port 1 with external pump filtering.

The cavity was characterized by net anomalous dispersion. The passive fibre had second order dispersion of 0.011 ps/(nm m) at 1450 nm, while the active fibre dispersion, estimated from Kelly sidebands, was −0.006 ps/(nm m) and thus slightly normal. Combined, the fibres contributed 0.10 ps/nm of double-pass anomalous dispersion. From one end, the cavity was terminated to a pair of reflection gratings and a silver mirror. This free space part increased the anomalous dispersion by 0.24 ps/nm. The other cavity elements had negligible dispersions. Thus, the total double-pass cavity dispersion amounted to 0.34 ps/nm.

### Description of the laser operation

**Figure 2:**
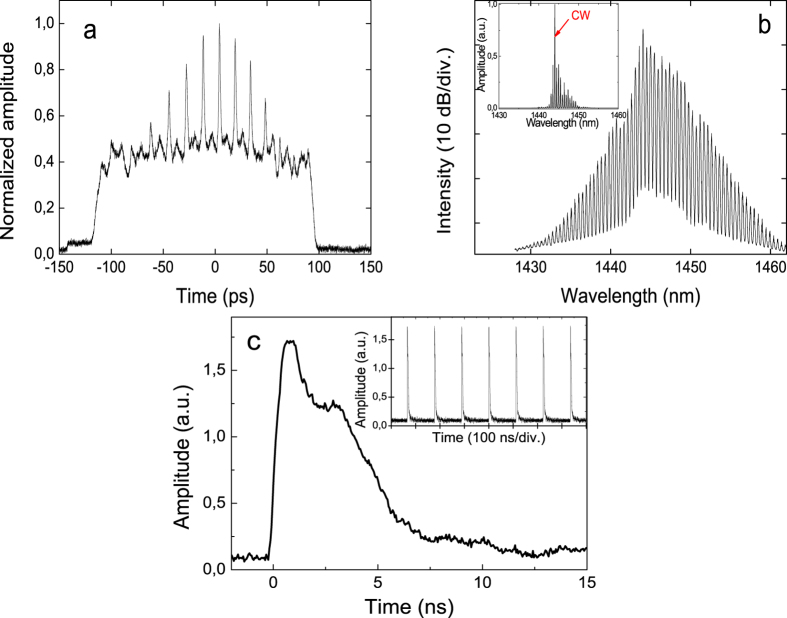
Infiltrated bound-bunch solitons mode. (**a**) – autocorrelation trace; (**b**) – optical spectrum in logarithmic and linear scale (inset); (**c**) – oscilloscope picture of the pulse train.

The laser displayed two types of soliton dynamics: dual bound-bunch solitons and pure bunch of solitons. Most commonly, the laser operated in infiltrated soliton bunching and bound soliton mode. The nature of this operation can be seen in the autocorrelation trace in Fig. 2a. The trace contains both a large pedestal and numerous narrow peaks with equal, 15.4 ps, spacing. We made sure that the extra pump light did not affect the autocorrelation or any other measurement. In particular, the pedestal in the autocorrelation was not visible when the laser was operated in CW mode below the mode-locking threshold. The pedestal is caused by the solitons within the bunch moving too fast for the autocorrelator to resolve them while the peaks confirm the simultaneous existence of stable bound solitons in the cavity. Four peaks can be seen on the left side of the highest peak. Even though only three peaks are easily resolved on the right side, the autocorrelation of bound solitons should always contain an odd number of peaks and the expected location of the fourth peak coincides with a dip in the signal that could be due to an impurity in the autocorrelator optics. Therefore, it is plausible that the total number of peaks in the autocorrelation trace should be nine, which corresponds to a bound state consisting of five solitons. It should be noted that this estimation has been based on the number of pulses whose amplitude exceed the pedestal’s amplitude, and the real number of solitons in the bound state can be higher. The obtained pulse duration was 1.89 ps (sech^2^ approximation) and the estimated 3 dB spectral width was 1.2 nm. The resulting time-bandwidth product 0.33 reveals almost transform-limited pulses delivered at this laser configuration.

The spectrum of the combined operation in Fig. 2b is deeply modulated with up to 30 dB extinction ratio between the peaks and the troughs. The reason for this is that the acousto-optic frequency shifter acts as a polarization comb filter. Even though the polarization dependent loss in the acousto-optic modulator is rather small, it can filter out one polarization component over multiple cavity roundtrips. The modulation period in Fig. 2b is about 0.47 nm or 68 GHz and agrees very well with the theoretical value of 0.5 nm based on the birefringence of the frequency shifter calculated from^25^


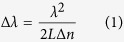


where Δ*λ* is the modulation period, *λ* is the central wavelength, L is the crystal length and Δ*n* is the difference of the refractive indexes between the slow and the fast modes. Because 68 GHz x 15.4 ps = 1.0472 ≈ 1, the spectral modulation indicates a non-random phase relationship over successive round trips. The polarization can also be tuned to obtain spectral modulation where half of the peaks are suppressed and the modulation period is effectively 0.94 nm or 136 GHz. In that case, the temporal separation of the peaks in the autocorrelation is half of its aforementioned value, 7.7 ps. The spectrum contains also strong CW, which is clearly seen in the linear scale (inset of Fig. 2b).

In the oscilloscope trace (Fig. 2c) (measured by Agilent Infinium 2.5 GHz oscilloscope and Thorlabs DET01CFC 1.2 GHz photodetector), the pulse train contains bunches of pulses separated by the cavity round trip time. Each bunch has a sharp leading edge and a slowly decaying trailing edge. The FWHM width of the bunch is about 5 ns at 1.6 W pump power and decreases if the pump power is decreased. The bunch also “breathes”, i.e. its width oscillates in time. RF spectra can be found in Supplementary Fig. S1.

**Figure 3:**
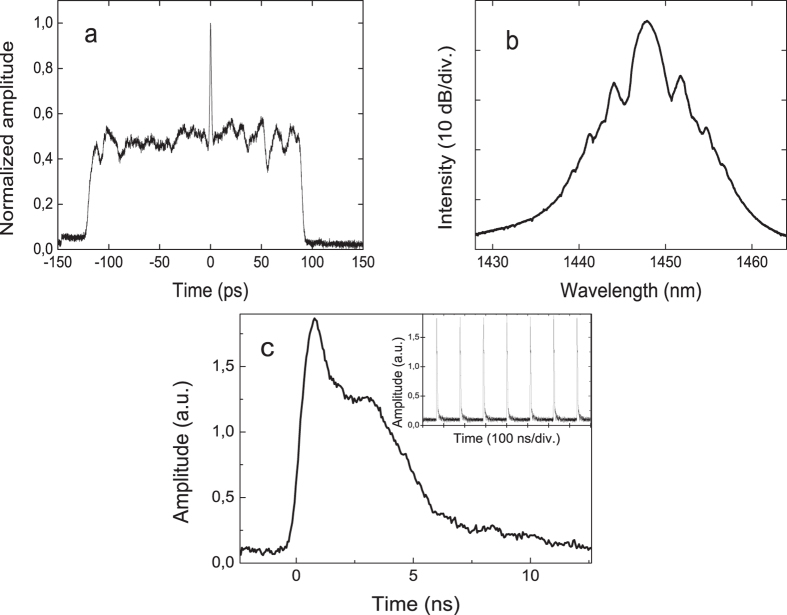
Pure bunch of soliton mode. The measured laser output parameters of the state: (**a**) autocorrelation trace, (**b**) optical spectrum and (**c**) oscilloscope picture of the pulse train.

By adjusting the polarization controllers the soliton regime can be switched from infiltrated to pure soliton bunching without any bound soliton component. In the autocorrelation (Fig. 3a), the pedestal caused by randomly moving pulses is still present, but instead of multiple peaks there is only a single coherence peak. The spectrum (Fig. 3b) is smooth with no modulation but contains greatly broadened Kelly sidebands. The pulse width was estimated as 1.91 ps and FWHM of the optical spectrum was equalled to 2 nm, yielding a time-bandwidth product of 0.52. The pulse train in the oscilloscope (Fig. 3c) looks similar to the case of combined bunch and bound states operation. Both soliton bunching and the infiltrated regime were observed at a wide range of pump powers from 0.4 W to 1.6 W and measured with the maximum pump power of 1.6 W. The laser operation was stable in the absence of polarization alteration.

It should be noted that pedestal-free bounded soliton operation could not be possible to achieved in the current setup. However, this operation appears as soon as total cavity dispersion has been decreased to 0.1 ps/nm. The details of pedestal-free operation in the similar cavity can be found in Supplementary Fig. S2.

### Computational model

**Figure 4:**
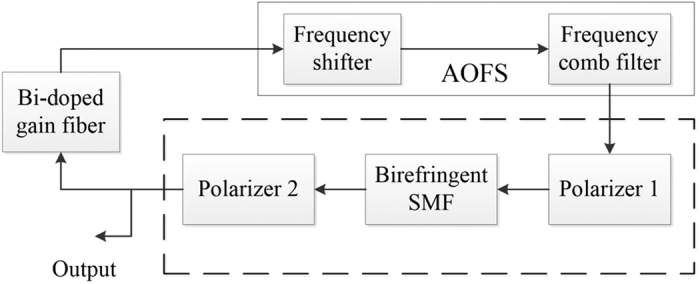
The principle scheme of the computational model. AOFS-acousto-optic frequency shifter, SMF – single mode passive fibre. Passage of elements united by a dashed line describes the propagation of radiation in the passive fibre components.

In this section we consider the computational model of the investigated Bi-doped fibre laser. We note that the target of the simulation is only a qualitative explanation of the main results of the experiment. The proposed model is similar to the one previously discussed in detail in refs 26,27. The model includes a consistent description of the radiation propagation in active and passive fibres according to the Ginzburg-Landau and coupled nonlinear Schrodinger equations (NSEs) and passing lumped elements: an acousto-optic frequency shifter (AOFS), polarizers, output coupler and etc. The principle scheme of the model is shown in Fig. 4. It should be noted that the goal of the modelling was not a literal replication of the experimental setup, but rather the investigation of the physical nature of the observed soliton groups. Therefore, each element of the model is essentially responsible for some key physical effects, for example, the effect of output coupler includes all linear losses in the cavity, AOFS operation is divided into proper frequency offset and the effect of a birefringent filter; complex process of radiation propagation in the birefringent fibre sections of the experimental scheme is reduced to a simplified nonlinear polarization rotation (NPR) action, operating in the system of the polarizers and the birefringent fibre^28^, etc. A further simplification is also due to the fact that the dispersion effects associated with diffraction gratings are not considered separately, their dispersion distributed between the fibre sections. A detailed description of the model is given in the “Methods” section.

**Figure 5:**
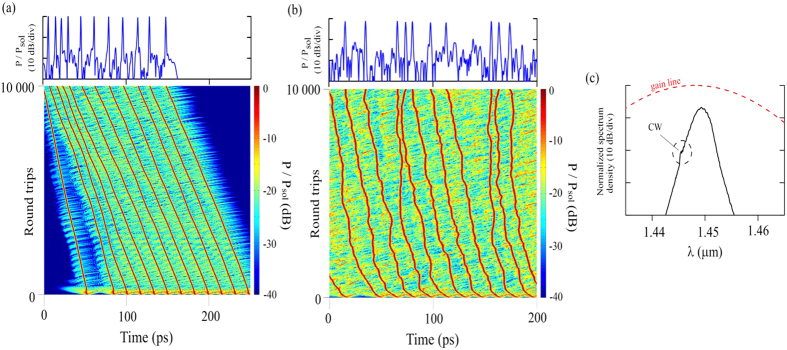
The formation of solitons from the initial noise and their dynamics under the influence of AOFS. (**а**) An example of formation and evolution of solitons in the proposed model under the influence of frequency shifting. On top the final pulse distribution is shown. (**b**) The same with the introduction of a CW radiation source with constant amplitude but with random phase. (**c**) The spectrum of solitons from (**b**), the gain line is also shown.

**Figure 6:**
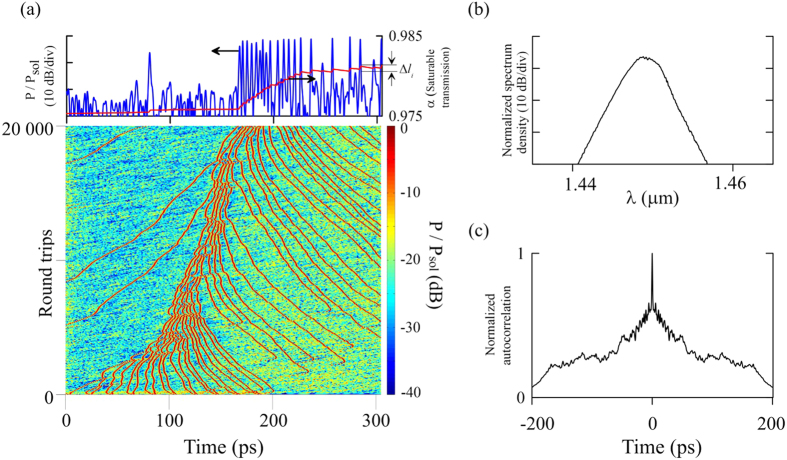
Formation of the soliton bunch in the presence of saturable absorption. (**a**) evolution of the soliton bunch. The pictures on the top show the final arrangement of the pulses and the level of transmission *α*, defined by the saturable absorption 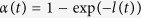. (**b**) Spectrum and (**c**) autocorrelation of soliton bunch.

**Figure 7:**
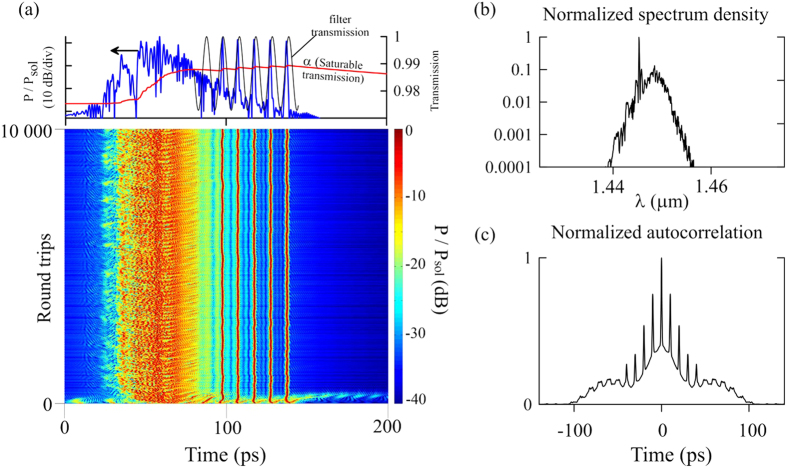
Formation of pulse group defined as “infiltrated bunch “ in the model with included polarization comb filter. (**a**) formation of infiltrated bunch. The top picture shows the final arrangement of the pulses, the transmission level 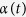 determined by saturable absorption, and transmission function of polarization comb filter 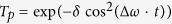. (**b**) Spectrum and (**c**) autocorrelation of bound solitons and bunch coexisting in the cavity.

The proposed model allows to profoundly describe the main features of the collective soliton dynamics in the experimentally studied cavity. We emphasize the main points, which are illustrated in Figs 5, 6, 7. Initially we exclude the effect of slow saturable absorption and polarization comb filter from the model, then subsequently we “turn on” these effects and consider their impacts. The simulation results presented in Fig. 5 demonstrate the formation of solitons from the initial noise and their dynamics under the influence of AOFS. It is known, that the frequency shift is a discriminating factor distinguishing solitons from low-amplitude radiation in nonlinear systems with limited gain bandwidth^29^,^30^. Nonlinear self-phase modulation (SPM) compensates for the frequency offset originated from AOFS and provides the stability of soliton pulse, whereas any nonsoliton components are quickly filtered. If the pulses are spaced far apart, the interaction between them is only the competition for the gain, whereby all pulses have the same carrier frequencies and peak powers. If the pulses are in close proximity, the AOFS action weakens direct inter-pulse interaction due to the linear growth of the phase difference between these pulses. Evidently a rapidly rotating phase difference between the pulses averages their net attraction and repulsion to zero^29^. These effects are illustrated in Fig. 5a that shows the high absorption of resonance dispersive radiation and suppression of direct interaction between close pulses.

Many real laser systems possess CW lasing component coexisting in cavity with solitons^31^,^32^ and the experimental results of the current work show that the investigated laser based on a Bi-doped fibre also exhibits similar behaviour (Fig. 2b). The interaction between solitons and the frequency-shifted CW component causes a change in the soliton frequencies and distortion of their trajectories. To account for this effect, a source generating a low-amplitude CW radiation was additionally introduced in the proposed model at each round trip of the cavity. The amplitude and frequency of the source are constant but the phase is periodically randomly altered. An example simulation is presented in Fig. 5b. Our simulations show that the AOFS cannot stabilize the soliton trajectories and suppress the inter-pulse jitter anymore when the level of CW component is rather high. In that case, CW radiation of interacting with solitons changes their frequencies and phases and randomizes trajectories. A similar change in soliton group dynamics can also occur without the introduction of an additional CW source, if the gain bandwidth is increased. In this case, filtering effect of resonant dispersive radiation is reduced and chaotic dynamics arises from the interaction between each soliton and dispersive waves excited by other pulses in the cavity. FSF laser pulses have a standard soliton spectrum (Fig. 5c), in this case we note that after averaging CW component in the spectrum hardly noticeable because of the periodically changing random phase.

At the next step, we consider the changes in soliton group dynamics including the relaxing slow saturable absorption in the model. It is important that the introduction of slow saturable absorption creates a new type of dynamics – the collective dynamics associated with the formation of the pulse groups. In this case, the absorption is time-dependent and the absorbance at the pulse leading edge is different from the absorption at the trailing edge by the amount 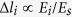 (Fig. 6). As a result, the pulse envelope is shifted to the region of lower absorption and the *i*^th^ soliton experiences an additional velocity drift proportional to Δ*l*_*i*_ in the direction of *t* → ∞. How soliton drift velocity changes under the influence of saturable and relaxing dissipative parameters is considered in greater detail in refs 33,34. In this work, we concentrate on the features of group dynamics in the system with relaxing slow saturable absorption.

The analysis of the rate equation for saturable absorption shows that the change in absorption during the i^th^ pulse passing is proportional to the value of absorption at leading edge of the *i*^*t*h^ pulse *l*_*i*_: 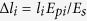. Consequently, for each forthcoming pulse in the group an increment to the velocity will be smaller than for the previous one. Thus, saturable absorption compresses the pulse group into a bunch. This process is illustrated in Fig. 6. It can be noted that the pulses are consistently included in the bunch, thereat, the drift velocity is determined not only by saturable absorption, but also by the competition for saturated gain. This competition is global for the whole soliton group, any local variation in radiation energy leads to the change of energy of every single pulse.

At the tight approach of the pulses in the bunch, the attractive force is counterbalanced by repulsion caused by direct interpulse interaction or interaction with the dispersive waves. These interactions depend on the phase difference between the interacting pulses. As a result, the pulse dynamics in the bunch is randomized and interpulse distances vary chaotically. In the experiment, this kind of dynamics is characterized by a wide pedestal in the autocorrelation function of the soliton assembly (Figs 2a and 3a) which appears because of averaging in the autocorrelator. Chaotic changes of interpulse distances lead to fluctuations in the bunch size, which can be observed as “bunch breathing”.

Attention is also drawn to the area of low absorption directly behind the bunch, where continuum periodically “breaks”, i.e. there is a generation of new pulses from an unstable non-soliton component. Later, the bunch approaches and merges with the new pulse due to the difference in the drift velocities. At the same time, the single pulses are periodically damped on the leading edge of the bunch in the area of increased absorption due to the competition for the saturated gain. This complex phenomenon can be observed in the experiment as a “soliton rain” – individual pulses periodically merge with the bunch. On the resulting bunch simulation spectrum (Fig. 6b), the CW component is not visible. This is, in particular, due to the strong dispersive background radiation generated in the area of low-saturated absorption. Autocorrelation of the simulated bunch (Fig. 6c) is similar to the experimental – by averaging effect the pulses moving relative to each other form a uniform image with broad pedestal.

Next, we consider a model with a polarization comb filter. As mentioned in Methods, the filter is introduced phenomenologically by using a transfer function of the form 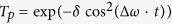. The modulation period Δ*ω* is determined by the birefringence of AOFS as 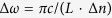 (Equation (1) rewritten through frequencies). The modulation depth *δ* > 0 characterizes the orientation of the fast and slow axes of the AOFS crystal relatively to the input linearly-polarized light and determines the level of filtering effect produced by the filter. Thus, *δ* = 0 corresponds to coincidence of the polarization plane of input light with the fast or slow axis of the AOFS crystal, while positive values of *δ* correspond to other orientations. The choice of this expression can be explained by the following considerations. The coefficient cos^2^ (Δ*ω* · *t*) is proportional to the response of the interferometer formed by birefringent crystal of AOFS, and the value of *δ* is determined by the coupling of the input linearly-polarized radiation into two channels of this interferometer. The exponential function is chosen just for convenient comparison with distributed parameters characterizing saturable absorption.

The simulation results using the filter are presented in Fig. 7(а). As it can be seen, the tightening of pulses into the bunch prevents by the action of the polarization filter, i.e., the drift velocity of some pulses proportional to jump of saturable absorption Δ*l*_*i*_ is insufficient for the pulse to leave “potential well” associated with the filter transmission peak. As a result, the pulse trajectory is stabilized and the distance between adjacent pulses becomes fixed. Moreover, the filter allows to clearly distinguish on the background radiation of CW component, through which the pulses interacting with each other to form a bound state of a constant phase difference^35^. This is proved, in particular, by modulation of the averaged soliton spectrum (Fig. 7(b)). On the leading edge of the bunch gain is insufficient for stable propagation of soliton. Amplification of background radiation can be observed there, as well as the birth and damping of unstable pulses and the generation of strong dispersion radiation. As in the experiment, this behaviour is manifested as the mixed autocorrelation function (Fig. 7(c), experimentally Fig. 2a) - sharp, stable peaks on top of a solid pedestal background. We propose to call this kind of a pulse group as “infiltrated bunch”.

## Discussion

Multi-soliton operation is an essential attribute of mode-locked fibre lasers operating in the net anomalous dispersion regime. All solitons generated in the non-PM fiber laser cavity are dissipative vector solitons due to the presence of intracavity losses, which are compensated for by the gain, and the absence of polarization discrimination in conventional fibres. The later fact means that solitons in the cavity have two orthogonal polarization components^36^,^37^,^38^. Under certain balance between linear and nonlinear birefringence in the cavity it can lead to the cases, when the soliton polarization state is locked, and two orthogonal polarization components propagate as entire unit, forming phase-locked or polarization-locked vector solitons. The parameters of vector soliton for orthogonal polarization modes can be different. In this paper, we focus on the investigation of a new type of dissipative soliton dynamics and leave the examination of vector nature of the solitons for future study.

Under the influence of dissipative effects featured in the cavity, different types of soliton interaction can occur resulting in the formation of multi-soliton complexes such as bound solitons, bunch of solitons and soliton rain. We demonstrate here a frequency-shifted feedback mode-locked bismuth-doped fibre laser with a novel form of nonlinear soliton dynamics. The acousto-optic frequency shifter starts and maintains mode-locking and, because of its strong polarization dependence, functions simultaneously as a polarization comb filter with a periodicity corresponding to the crystal properties. The Bi-doped fibre provides sufficient gain to generate a huge number of solitons in the cavity. Soliton group dynamics are determined mainly by the dissipative parameters of the system – saturated gain and slow relaxing saturable absorption. The action of these parameters leads to the pulse drift into the region of the maximum gain, thereat the saturated gain contributes to mutual repulsion of pulses and the formation of harmonic mode-locking regime; saturable absorption, on the contrary, provides a contraction of pulse groups into tight bunches^8^,^33^,^34^. The bunch build-up in the experiment shows the influence of slow relaxing saturable absorption in the Bi-doped fibre dominates over saturated gain. This conclusion, in particular, has been used in developing the laser model. The simulation results are in a qualitative agreement with the experiment, and allow to distinguish some correlation between the parameters of the system. Indeed, the number of pulses N in a bunch, and their energy is determined by the gain saturation. The strength of the bunch compression depends on the difference of the drift velocities of the pulses and, accordingly, the ratio of the saturable absorption level to its saturation energy *l*_0_/*E*_*s*_. On the leading edge of the bunch the compression is maximized, while near the bunch tail, together with an increase in the saturation the average distances between pulses increases. Absorption relaxation time *τ*_*s*_ determines the characteristic scale of the collective pulse dynamics. In a compact group, the distance between pulses *τ* ≪ *τ*_*s*_ varies randomly, as the strength of the interaction depends on the phase difference. Because of this, the width of the bunch constantly fluctuates. When the distance between the bunch and the individual pulses are of the order of *τ*_*s*_ and more, there is an attraction which does not depend on the phase difference, resulting in an effect of “soliton rain” - a smooth pulse and bunch approach. The pulse interaction with the dispersion radiation and CW component in addition is randomized group dynamics.

The main result of the work is the experimental discovery of a new kind of soliton group dynamics in a fibre laser. This type of operation features both a background of chaotic bunch dynamics and a pulse group with a constant interpulse spacing. The constant spacing is the result of interferometric filtering in the birefringent crystal in the AOFS, as indicated by the coincidence of the frequency modulation in the experimental spectrum and the theoretical modulation period of the birefringent filter. The simulation confirms that such groups can form on a permanent background of chaotic bunch and explains how they can be switched from the pure chaotic bunch regime into mixed mode of “infiltrated bunch” by the change of the coupling coefficient, which depends on the relative orientation of the crystal and the polarized radiation. In other words, polarization adjustment allows varying the depth of “potential wells” where the pulses are trapped. At the highest level of filtration, it is possible to establish the highest pulse approaching, even when the direct solitons interaction does not shift pulses beyond the “well” (in the experiment this corresponds to the pulse separation of 7.7 ps and a spectral modulation with a period of 0.94 nm). Moreover, the filter allows to clearly distinguish CW component, through which the pulses interacting with each other to form a bound state of a constant phase difference. By reducing the filtration effect, stationary groups with double the previous interpulse distance can be observed (in this case the corresponding pulse distance is 15.4 ps and modulation spectrum with a period of 0.47 nm). The pulse distance increase is explained by a decrease in the depth range of “potential well”, and the mutual pulses repulsion through the direct interaction. Finally, the effect of polarization comb filter can be “switched off”. In that case, the laser operates in a pure soliton bunch regime with only chaotically moving pulses.

## Methods

As in standard consideration Bi- doped gain fibre in the model is characterized by the following values – small signal gain *g*_0_, energy of gain saturation *E*_g_, gain bandwidth Ω_*g*_. We believe that variations of gain during cavity roundtrip time are very small, so the gain can be averaged, therefore in the simulation window it is assumed to be constant 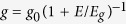, where *E* is the total radiation energy. Dispersion and nonlinear fibre characteristics are indicated by group-velocity dispersion *β*_2g_ and parameter of Kerr nonlinearity *γ*_*g*_. Besides of that, Bi- doped fibre as active medium is distinguished by a significant slow saturable absorption specified as *l*. It is characterized by its initial level *l*_0_, saturation energy *E*_*s*_ and relaxation time *τ*_*l*_. Contrary to saturated gain we consider slow saturable absorption as a parameter, which can change during a single roundtrip of the cavity. The value of this parameter is determined by the standard rate equation. The main features of saturable absorption behaviour are stepwise drops 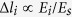 related to the passage of the *i*^th^ pulse in the cavity. Then these drops are altered by slow relaxations.

Action of acousto-optic modulator is introduced by the transfer function, indicating a spectrum shift on the frequency Ω_*f*_. To simplify the model, the total effect of polarization comb filter, accompanying the passage through the modulator is introduced phenomenologically, i.e. via transfer function of comb-like filter with appropriate frequency modulation period Δ*ω* and the modulation depth *δ* depending on the relative orientation of the modulator with respect to the polarization axes.

Propagation of radiation in the birefringent single mode fibre is described by using two coupled nonlinear Schrodinger equations in a similar way as in ref. 39. Two coupled modes are created at a polarizer which divides the radiation into two orthogonally polarized components. After propagation in SMF a polarization analyser, on the contrary, combines these components into linearly polarized light. Orientation of the polarizers in relation to the axes affects the nonlinear radiation loss in the cavity, which can lead to the change of observable regimes. The output coupler is characterized by constant real transmission coefficient *T*<1. We used small-amplitude noise as the initial condition of the simulation window, which represents a certain temporal domain of the cavity with the size of about hundreds of picoseconds. We should note that frequency shifted pulses can leave the resting simulation window. In order to avoid this problem, the whole window is periodically shifted.

The propagation of radiation in active Bi-doped fibre was described by the nonlinear Ginzburg-Landau equation taken in a form of


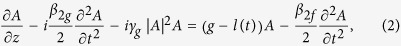


using the following variables and parameters: *A*(z,*t*) is a slowly varying amplitude of the field, z is the propagation distance along the active fibre, *β*_2*g*_ is the group velocity dispersion (GVD) and *γ*_*g*_ – Kerr nonlinearity coefficient of the active fibre. The saturated gain *g* can be averaged in simulation window and it is expressed as


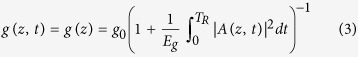


where *g*_0_ is small signal gain and *E*_*g*_ – gain saturation energy. *T*_*R*_ is the size of the simulation window in our case. The gain spectral filtering employs the parabolic approximation 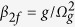, where Ω_*g*_ is half gain bandwidth. In our consideration we neglect linear losses in the Bi-doped fibre compared to saturable absorption *l(t*). It is determined by standard rate equation


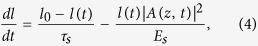


where *l*_0_- unsaturated losses, *E*_*s*_ – absorption saturation energy and *τ*_*s*_- relaxation time.

The propagation of radiation in passive single mode fibre was described by two coupled NSE^25^


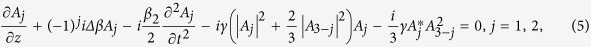


where *A*_*j*_ are the amplitudes of the of the field along the two orthogonal polarized modes, *β*_2_ is the GVD.*γ* is Kerr nonlinearity coefficient of the fibre and Δ*β* is wavenumber difference related to the birefringence of the fibre. Linear losses are assumed to be part of output coupler losses. Two orthogonal polarized modes are defined at fibre input as 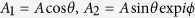. The analyser mixes the two components into a single component 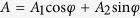. Solution of the equations (2) and (5) was performed by standard Fourier split step method.

Impact of discrete cavity elements – acousto-optic frequency shifter (AOFS), output coupler – were accounted by using a transfer function of each element. The transfer function of the AOFS was applied in spectral domain 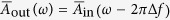, where Δ*f* is the frequency of the active modulator. Its value was taken as frequency net partition size. Impacts of the AOFS as interferometer and output coupler were applied in the temporal domain 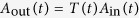, where for interferometer 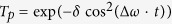, and for output coupler 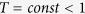. In simulations we assume *T* = 0.95.

In simulations as in experiment we suggest that the GVD of Bi-doped fibre is normal (*β*_2*g*_>0) and the GVD of single-mode fibre is anomalous (*β*_2*g*_<0). The GVD values relate as *β*_2*g*_ = −*β*_2_/2 and the total GVD of the cavity *β*_2*0*_ = *β*_2*g*_+*β*_2_ is anomalous. The nonlinearities of Bi-doped and single-mode fibres are assumed equal *γ*_*g*_ = *γ* = *γ*_*0*_. The power in Figs 5, 6, 7 is measured in soliton units 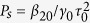 (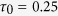
ps).

In the Table 1 we introduce the values of the parameters used in simulations. They are related to the full roundtrip of the cavity.

## Additional Information

**How to cite this article:** Rissanen, J. *et al*. Infiltrated bunch of solitons in Bi-doped frequency-shifted feedback fibre laser operated at 1450 nm. *Sci. Rep.* **7**, 44194; doi: 10.1038/srep44194 (2017).

**Publisher's note:** Springer Nature remains neutral with regard to jurisdictional claims in published maps and institutional affiliations.

## Supplementary Material

Supplementary Information

## Figures and Tables

**Table 1:** Laser cavity parameters used in the numerical simulation.

Parameter	Value	Parameter	Value
*E*_*g*_	675 pJ	*E*_*s*_	250 pJ
*g*_0_	2 dB	*l*_0_	−0.11 dB
*γ*_0_	0.05 W^−1^	*τ*_*s*_	250 ps
*β*_20_	−0.05ps^2^	Ω_g_	8 ps^-1^
*θ*	*π*/30	*ϕ*	0−π/15
*φ*	−*π*/4.15	2*π*Δ*f*	0.015 ps^-1^
Δ*ω*	0.6 ps^-1^	*δ*	0–0.025
