# Supplementary material for: Infiltrated bunch of solitons in Bi-doped frequency-shifted feedback fibre laser operated at 1450 nm
Source: Sci Rep. 2017 Mar 10;7:44194. doi: 10.1038/srep44194 (PMC5345058; doi:10.1038/srep44194)
Supplement: Supplementary Information [file srep44194-s1.pdf]

**Supplementary information**

# Infiltrated bunch of solitons in Bi-doped frequency-shifted feedback fibre laser operated at 1450 nm

Joona Rissanen[1], Dmitry A. Korobko[2], Igor O. Zolotovsky[2], Mikhail Melkumov[3], Vladimir F. Khopin[4], and Regina Gumenyuk[1]*

[1]Optoelectronics Research Centre, Tampere University of Technology, 3 Korkeakoulunkatu, 33720 Tampere, Finland,

[2]Ulyanovsk State University, 42 Leo Tolstoy street, 432017, Ulyanovsk, Russia

[3]Fiber Optics Research Center, Russian Academy of Sciences, 38 Vavilov Street, 119333 Moscow, Russia

[4]Institute of Chemistry of High-Purity Substances, Russian Academy of Sciences, 49 Tropinin Street, 603600 Nizhny Novgorod, Russia

**Corresponding author: regina.gumenyuk@tut.fi*

**RF spectra of the infiltrated bunch state**

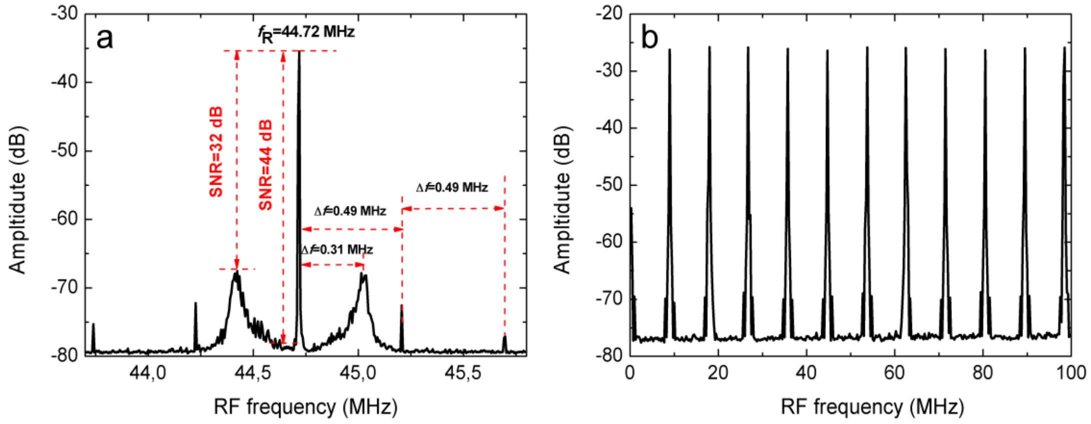


Fig. S1. **RF spectra of the infiltrated bunch operational state of the laser**. a – 1st harmonics of the pulse; b – pulse train with 100 MHz span range.

The measured RF spectra of the infiltrated bunch of solitons state are presented in Fig. 2. The spectrum shown in Fig. 2a was measured with a span of 2 MHz and 1 kHz resolution. The signal to noise ratio was equal to 44 dB for the central peak. RF spectrum contains several symmetrical with respect to the central peak satellites. One pair is separated from the central peak by 0.31 MHz. It is

characterized by relatively broad spectrum. Two others are equidistantly disposed peaks with separation of 0.49 MHz. The spectra of these pairs are sharp and with a small amplitude. Fig. 2b shows number of subsequent harmonics within 100 MHz span range with 10 kHz resolution. The train does not reveal any variation indicating well-operated mode-locked regime.

RF spectra were measured with polarization controller at the output pigtail, which established the linear polarization state at the careful adjustment of the paddles. In this case RF spectra can also provide the information regarding polarization state evolution of the pulse passed through polarizing element. As it was shown by B. C. Collings et al.[1] the sidebands around the harmonics of the repetition rate frequency are the signature of the evolution of the pulse polarization transforming into amplitude modulation at the output of the polarizer. The rate of polarization evolution is expressed in polarization evolution frequency (PEF). When polarization is locked, PEF=0. When polarization perturbation of soliton occurs, the PEF takes value other than zero. As it can be seen from the Fig. S1a the symmetrical sidebands stated apart of the central peak with 0.31 MHz can be classified as PEF and, thereby, reveal that the polarization state is not locked. The satellites around the central peak are characteristics feature of group-velocities-locked vector soliton (GVLVS) types, but side peak are usually narrow for the locked operation. The broad spectra of PEF refer to chaotic movement and generation of solitons on the leading edge of the bunch (See Fig. 7a).

The nature of two equidistant pairs with 0.49 MHz separation relates to CW component, distinguished by the filter, and its harmonics. It can be considered as analog of Kelly sidebands in soliton spectrum. The frequency difference in 0.49 MHz corresponds to shifting of CW component with respect to the bunch of solitons.

**Pedestal-free bounded soliton state**

We investigated whether we could eliminate the soliton bunching and the pedestal in the autocorrelation altogether. This was found impossible with the original high cavity dispersion and high pump power. However, we were able to obtain bound state operation without soliton bunching by replacing the grating pair with a butt-coupled silver mirror, which reduced the anomalous dispersion to 0.10 ps/nm, and using relatively low pump power of roughly 0.8 W. This is in accordance with an earlier study[2], which has shown that high dispersion and nonlinearity tend to favor soliton bunch operation over bound solitons. Once again, the nature of the operation is best seen in the now pedestal-free autocorrelation (Fig. S2a). The existence of one side maximum on either side of the main peak indicates the presence of a bound soliton pair. The spectrum resembles a normal soliton spectrum in a fiber laser with its sharp sidebands, though the center of the spectrum is weakly modulated because of the bound soliton operation (Fig. S2b). There is also a weak, unstable CW component near the center of the spectrum. Its existence is explained by the pulse train in Fig. S1c, which depicts a soliton rain. The peculiar thing about this soliton rain is that the smallest unit is not a single pulse but a bound soliton pair as evidenced by the autocorrelation. In fact, we have never observed normal single or multi-pulse operation in this laser; the output always consists of grouped solitons as soliton bunches, bound solitons or both. The pulse separation was equaled to 8 ps, whereas the pulse duration was 0.81 ps, what can identify them as tightly-bound solitons[3]. The period of spectral modulation was 1 nm. The modulation amplitude was equal to 2.25 dB. The lower modulation amplitude compare to regime in Fig.2 tells that the phase relationship between pulses in the bound state is deviated from $\pi/2$ evolving within the number of several round trips, but the pulse distance is still constantly-fixed[4]. The FWHM of the spectrum was 3.2 nm. The time-bandwidth product corresponded to 0.37 indicating nearly-chirp-free pulses.

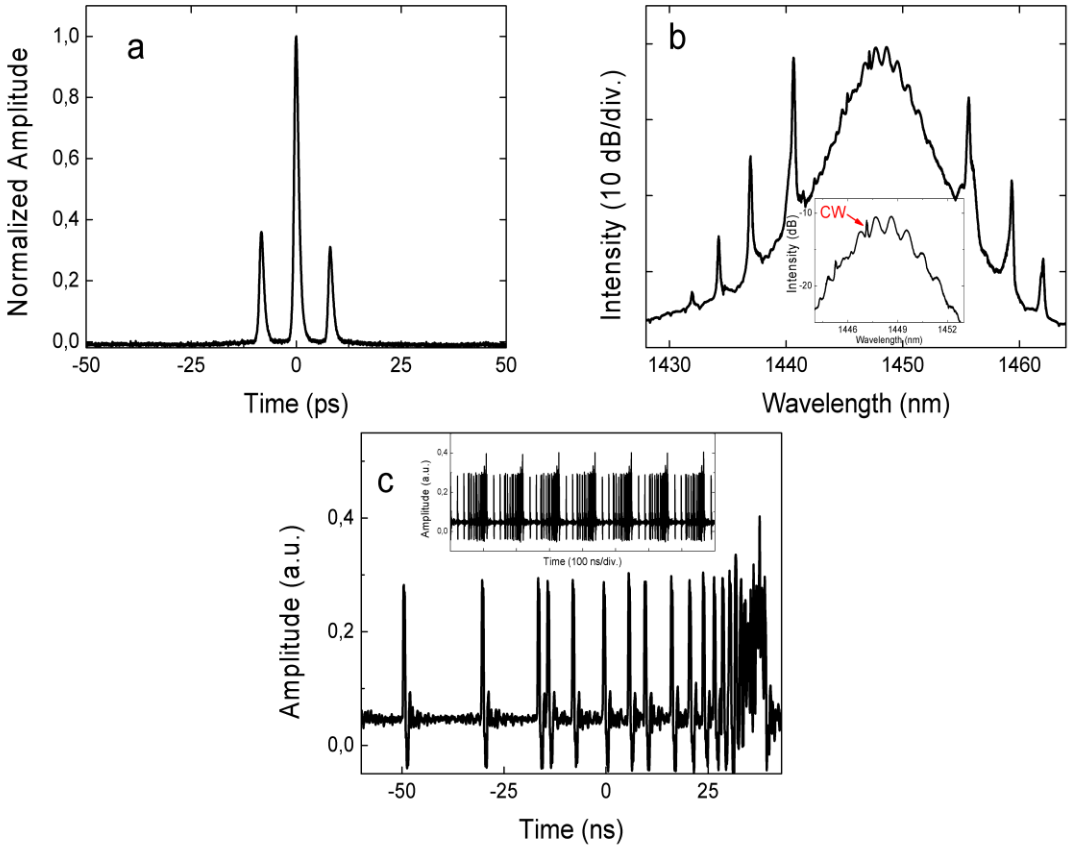


Fig. S2. Pedestal-free bounded solitons state. a – autocorrelation trace; b – optical spectrum (in the inset - the scaled spectrum top part), c – oscilloscope picture of the pulse trace.

**References**


1. Collings, C., et al. Polarization-locked temporal vector solitons in a fiber laser: experiment. *J. Opt. Soc. Am. B* **17**, 354-365 (2000)
2. Gumenyuk, R. & Okhotnikov, O. G. Multiple solitons grouping in fiber lasers by dispersion management and nonlinearity control. *J. Opt. Soc. Am. B* **30,** 776 (2013).
3. Wu, X., Tang, D.Y., Luan, X.N. & Zhang, Q. Bound states of solitons in a fiber laser mode locked with carbon nanotube saturable absorber. *Opt. Commun*. **284**, 3615-3618 (2011).
4. Ortaç, B. et al. Observation of soliton molecules with independently evolving phase in a mode-locked fiber laser. *Opt. Lett*. **35**, 1578-1580 (2010).
